# Acetylated Hyaluronic Acid: Enhanced Bioavailability and Biological Studies

**DOI:** 10.1155/2014/921549

**Published:** 2014-07-08

**Authors:** Carmela Saturnino, Maria Stefania Sinicropi, Ortensia Ilaria Parisi, Domenico Iacopetta, Ada Popolo, Stefania Marzocco, Giuseppina Autore, Anna Caruso, Anna Rita Cappello, Pasquale Longo, Francesco Puoci

**Affiliations:** ^1^Department of Pharmacy, University of Salerno, Via Giovanni Paolo II 132, 84084 Fisciano, Italy; ^2^Department of Pharmacy, Health and Nutritional Sciences, University of Calabria, 87036 Arcavacata di Rende, Italy; ^3^Department of Computer Engineering, Modeling, Electronics and Systems, University of Calabria, 87036 Rende, Italy; ^4^Department of Sciences, University of Salerno, Via Giovanni Paolo II 132, 84084 Fisciano, Italy

## Abstract

Hyaluronic acid (HA), a macropolysaccharidic component of the extracellular matrix, is common to most species and it is found in many sites of the human body, including skin and soft tissue. Not only does HA play a variety of roles in physiologic and in pathologic events, but it also has been extensively employed in cosmetic and skin-care products as drug delivery agent or for several biomedical applications. The most important limitations of HA are due to its short half-life and quick degradation *in vivo* and its consequently poor bioavailability. In the aim to overcome these difficulties, HA is generally subjected to several chemical changes. In this paper we obtained an acetylated form of HA with increased bioavailability with respect to the HA free form. Furthermore, an improved radical scavenging and anti-inflammatory activity has been evidenced, respectively, on ABTS radical cation and murine monocyte/macrophage cell lines (J774.A1).

## 1. Introduction

Hyaluronic acid (HA), the main component of the glycosaminoglycans, is a linear biodegradable polymer with high molecular weight consisting of disaccharide units of* N*-acetylglucosamine and* D*-glucuronic acid, connected alternately by *β* 1-3 and 1-4 glycosidic bonds. HA is naturally present in almost all body fluids and tissues such as the synovial fluid, eye vitreous humor, connective, epithelial, and neural tissues and plays important biological functions in wound healing regulating cell adhesion, motility, differentiation, and proliferation. HA assists the early phases of the inflammatory process, improving cell infiltration and facilitating an increase in proinflammatory cytokines and, afterwards, the free radical scavenging and antioxidant characteristics of HA allow suppressing the inflammatory response during the healing process [1]. This dual role played during the inflammation phases depends on HA molecular mass indeed; in its native state, it generally exists as a high-molecular-mass polymer whereas, under inflammation, HA is more polydisperse, with a preponderance of lower-molecular-mass forms [2]. Besides, several studies shed light on other key roles played by HA in influencing cellular processes, for instance, morphogenesis, cancer progression, and metastasis [3]. Indeed, many HA fragments have been found in a wide range of carcinomas, lymphomas, melanocytic, and neuronal tumors; these fragments exhibit properties, not normally found in the native HA polymer, whose effects depend on the molecular size as, for instance, angiogenics or growth suppressing. The altered HA metabolism and the amount of itself in the tumor stroma or in the neoplastic cell compartment are strictly associated with invasion and local or distant metastases, impacting on the overall outcome [4]. HA and its derivatives have been also employed as anticancer drug carriers because of their ability to be recognized by specific cellular receptors overexpressed on tumor cells membrane [5, 6].

Although properties such as high biocompatibility, biodegradability, high hydrophilicity, and viscoelasticity led to considerable use of HA both in medicine and in cosmetics, in particular, in the treatment of joint problems and in the “tissue augmentation” [7], its solubility in water, rapid absorption, and short residence time* in situ* limit its application [8, 9]. Moreover, exogenous HA is quickly degraded* in vivo* (half-life of 12–24 h) by hydrolytic enzymes, that is, hyaluronidases (HAses). To ensure greater permanence* in situ*, the HA is generally subjected to chemical changes such as derivatization or, especially, cross-linking processes [10], which decrease the solubility in water and increase its resistance to enzymatic degradation. Currently on the market, there are several “stabilized” (cross-linked) and biocompatible gels based on HA [8, 9] and the research is continuously engaged in the development of new derivatives that have advantages over those already in use, in terms of both degradation times and native polymer biocompatibility conservation.

**Figure 1:**
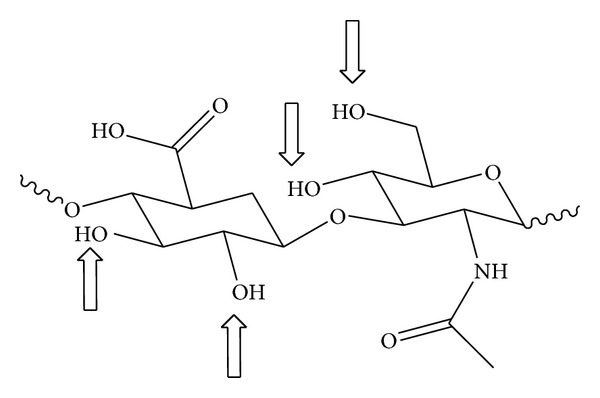
Functional groups of HA subjected to chemical modification.

In this paper, we reported the preparation of the acetyl ester of HA (HA-Acet) (Figure 1), with the aim to prolong the effect and improve its radical scavenging, antioxidant properties and bioavailability* in vitro. *We have also evaluated the HA and HA-Acet cytotoxicity in three cellular lines, that is, murine monocyte/macrophage cell line (J774A.1), murine fibrosarcoma cells (WEHI-164), and human epithelial kidney cells (HEK-293), and, after that, the HA-Acet inhibition of NO release from J774A.1 murine macrophages has been studied in comparison with the free HA form. 

## 2. Material and Methods

### 2.1. Chemistry

Unless stated otherwise, all reagents and compounds used were obtained from Sigma-Aldrich (Milan, Italy). The synthesis was made using sodium hyaluronate and its molecular weight (90 kDa) was determined by GPC (gel permeation chromatography). GPC analysis of the sample was made at 35°C using a tool, equipped with UV detector, refractive index detector, and a set of four PPS columns (made of polystyrene) having pore dimensions, respectively, of 10^5^ Å, 10^4^ Å, 10^3^ Å, and 10^2^ Å and particles size of 10 *μ*m. It was used as a solvent tetrahydrofuran at a rate of flow of 1.0 mL/min. For the determination of the molecular weight a calibration curve was obtained. The progress of the reaction was controlled by thin-layer chromatography (TLC), performed on a 0.25 mm layer of silica gel 60 PF254 Merck. The final product (MW 94 kDa) was purified by column chromatography with silica gel (Merck silica gel) and characterized by ^1^H NMR (300 MHz) and the spectrum was recorded on Bruker 300 spectrometer.

### 2.2. Procedure for the Acetylation of Hyaluronic Acid (HA-Acet)

**Scheme 1:**
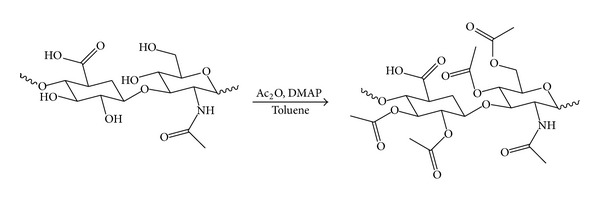
Acetylation of hyaluronic acid.

To a stirred cold solution (0°C) of 500 mg of sodium hyaluronate in 10 ml of toluene were added a catalytic amount of 4-dimethylaminopyridine (DMAP) and an excess of acetic anhydride. The mixture was stirred at reflux, under nitrogen, for 24 hours and then concentrated under reduced pressure. The solid residue was purified by silica gel chromatography using dichloromethane and methanol (9 : 1) as eluent, obtaining the pure compound as white solid [11–14] (Scheme 1). ^1^H NMR (CDCl_3_): *δ* 2.10 (s, 9H, 3 OCOC*H*_3_); 2.20–2.50 (m, 6H, CH_2_OCOC*H*_3_, HNCOC*H*_3_); 4.10–4.40 (m, 4H, 2 CH_2_); 4.45–5.50 (m, 9H, C*H*); 6.90–7.10 (br, 1H, O*H*); 8.10–8.50 (br, 1H, N*H*).

### 2.3. Determination of Scavenging Effect on ABTS Radical Cation

The scavenging activity of native HA and HA-Acet towards the hydrophilic ABTS (2,2′-azinobis-(3-ethylbenzothiazoline-6-sulfonic acid)) radical cation was assessed according to the literature with slight modifications [15]. ABTS was dissolved in water to a 7 mM concentration; radical cation (ABTS^•+^) was produced by reacting ABTS stock solution with 2.45 mM potassium persulfate (final concentration) and allowing the mixture to stand in the dark at room temperature for 12–16 h before use. Because ABTS and potassium persulfate react stoichiometrically at a ratio of 1 : 0.5, this will result in incomplete oxidation of the ABTS. Oxidation of the ABTS commenced immediately, but the absorbance was not maximal and stable until more than 6 h had elapsed. The concentration of the resulting blue-green ABTS^•+^ solution was adjusted to an absorbance of 0.970 ± 0.020 at 734 nm. The radical was stable in this form for more than two days when stored in the dark at room temperature.

In the present study, 10 mg of each sample was mixed with 5 mL of ABTS radical solution. The mixtures, protected from light, were incubated in a water bath at 37°C for 5 min. The decrease of absorbance at 734 nm was measured at the endpoint of 5 min. The antioxidant activity was expressed as a percentage of scavenging activity on ABTS radical according to

(1) $$\begin{array}{c} \text{Inhibition}\left(\%\right)=\frac{A_{0}-A_{1}}{A_{0}}\times 100, \end{array}$$

where *A*_0_ is the absorbance of a standard prepared in the same conditions, but without any sample, and *A*_1_ is the absorbance of the hyaluronic acid samples. All samples were assayed in triplicate and data expressed as means (±SD).

### 2.4. Nitric Oxide Radical (NO^∙^) Scavenging Assay

The anti-inflammatory activities of native HA and HA-Acet were evaluated by performing the* in vitro *nitric oxide radical scavenging assay, in which NO^∙^ generated from sodium nitroprusside (SNP) was measured spectrophotometrically according to the method reported in literature with slight modifications [16]. 10 mg of each sample was incubated with 1.0 mL of the reaction mixture, containing SNP (5 mM) in phosphate-buffered saline (pH 7.3), at 25°C for 3 h in front of a visible polychromatic light source (25 W tungsten lamp). The generated NO^∙^ radical interacted with oxygen to produce the nitrite ion (NO_2_^−^) which was assayed at 30 min intervals by mixing the incubation mixture with 1 mL of Griess reagent (1% sulfanilamide in 5% phosphoric acid and 0.1% naphthylethylenediamine dihydrochloride). The absorbance of the chromophore (purple azo dye) formed during the diazotization of nitrite ions with sulphanilamide and subsequent coupling with naphthylethylenediamine dihydrochloride was measured at 546 nm. The anti-inflammatory activity was expressed as a percentage of scavenging activity according to (1). Each experiment was performed in triplicate and the data presented as average of three independent determinations.

### 2.5. *In Vitro* Bioavailability Studies

*In vitro* bioavailability studies were carried out in simulated gastric and intestinal fluids by performing a slight modified version of the dialysis tubing procedure [17, 18] with the aim to simulate the oral intake of native and acetylated hyaluronic acid. The dialysis tubing method is characterized by two consecutive enzymatic digestions: pepsin and pancreatin digestion, respectively. These steps are described as follows. 

*Pepsin Digestion.* A 30 mg amount of each sample was mixed with 1.0 mL of a 0.85 N HCl solution containing 24000 U of porcine pepsin per mL. The obtained mixture was introduced into a dialysis bag (Spectrum Laboratories Inc., MWCO: 12–14,000 Dalton, USA), which was then carefully closed and immersed inside a flask containing 5 mL of a 0.85 N 60 HCl solution (pH 1.0). The flask was then incubated in a shaking water bath at 37°C to simulate the human body temperature conditions for 2 h. 

*Pancreatin Digestion.* At the end of the 2 h pepsin digestion, the dialysis bag was opened and 11 mg of amylase, 11 mg of esterase, and 1.3 mL of a 0.8 M NaHCO_3_ solution containing 22.60 mg porcine pancreatin/mL were added to the peptic digesta. After the digesta and enzyme solution were well mixed, the dialysis bag was sealed at each end with clamps 70 and placed in a flask with 5 mL of buffer solution at pH 7.0. The flask was incubated in the shaking water bath at 37°C for a further 4 h. After the pancreatin incubation time, the hydrolyzed hyaluronic acid was determined spectrophotometrically according to the literature [19]. Each experiment was performed in triplicate.

### 2.6. Cell Lines and Cultures

The murine monocyte/macrophage cell line (J774A.1), murine fibrosarcoma cells (WEHI-164), and human epithelial kidney cells (HEK-293) were obtained from American Tissue Culture Collection (ATCC). Dulbecco's modified Eagle's medium (DMEM), penicillin/streptomycin HEPES, glutamine, fetal calf serum (FCS), and horse serum were from Euroclone (Euroclone-Celbio, Pero, Milan, Italy). J774.A1 were grown in adhesion on Petri dishes and maintained at 37°C as previously described [20]. WEHI-164 and HEK-293 were maintained in adhesion on Petri dishes with DMEM supplemented with 10% heat-inactivated FCS, 25 mM HEPES, 100 u/mL penicillin, and 100 *μ*g/mL streptomycin.

### 2.7. Cell Viability Assay

J774.A1, WEHI-164, and HEK-293 (3.5 × 10^4^ cells/well) were plated on 96-well microtiter plates and allowed to adhere at 37°C in a 5% CO_2_ atmosphere for 2 h. Thereafter, the medium was replaced with 50 *μ*L of fresh medium and 75 *μ*L aliquot of serial dilution of each test compound was added and then the cells were incubated for 72 h. Serial dilution of 6-mercaptopurine (6-MP) was added, as reference drug. In some experiments, HA or HA-Acet were added only to J774A.1 macrophages for 24 h. Mitochondrial respiration, an indicator of cells viability, was assessed by the mitochondrial-dependent reduction of [3-(4,5-dimethylthiazol-2-yl)-2,5-phenyl-2H-tetrazolium bromide] (MTT) to formazan and cells viability was assessed as previously reported [21–28].

Briefly, 5 *μ*L of MTT (5 mg/mL) was added and the cells were incubated for an additional 3 h. Thereafter, cells were lysed and the dark blue crystals solubilised with 100 *μ*L of a solution containing 50% (v:v)* N*,*N*-dimethylformamide and 20% (w:v) SDS with an adjusted pH of 4.5 [29]. The optical density (OD) of each well was measured with microplate spectrophotometer (Titertek, Multiskan MCC/340) equipped with a 620 nm filter. The viability of each cell line in response to treatment with tested compounds and 6-MP was calculated as % dead cells = 100 − (OD treated/OD control) × 100. IC_50_ values (concentration that causes 50% growth inhibition) were determined [30].

### 2.8. NO_2_^−^ Release from J774A Cells

Nitrite content (NO_2_^−^), index of NO released by cells in the culture supernatant, was measured in J774A.1 cells. To stimulate nitric oxide (NO) release from macrophages,* E. coli* lipopolysaccharide (LPS, 6 × 10^3^ u/mL) was used [20]. Macrophages (3.5 × 10^4^ cells/well) were plated on 96-well microtiter plates and allowed to adhere at 37°C in a 5% CO_2_ atmosphere for 2 h. HA and HA-Acet (12.5–100 *μ*mol/L) were added for 1 h to cells and then coexposed to 1 *μ*g/mL LPS for further 24 h. NO_2_^−^ amounts were measured by Griess reaction. Briefly, 100 *μ*L of cell culture medium was mixed with 100 *μ*L of Griess reagent—equal volumes of 1% (w:v) sulphanilamide in 5% (v:v) phosphoric acid and 0.1% (w:v) naphthylethylenediamine-HCl—and incubated at room temperature for 10 min, and then the absorbance was measured at 550 nm in a microplate reader Titertek (Dasit, Cornaredo, Milan, Italy). The amount of NO_2_^−^ (as *μ*mol/L) in the samples was calculated from a sodium nitrite standard curve.

### 2.9. Data Analysis

Data are reported as mean ± standard error mean (s.e.m.) values of independent experiments, which were done at least three times, each time with three or more independent observations. Statistical analysis was performed by Student's *t*-test or analysis of variance test, and multiple comparisons were made by Bonferroni's test. A *P* value less than 0.05 was considered significant.

## 3. Results and Discussion

### 3.1. Evaluation of the HA and HA-Acet Antioxidant and Anti-Inflammatory Activity

In the aim to establish the antioxidant and anti-inflammatory activities of HA and HA-Acet, their reactivity towards ABTS and nitric oxide (NO^∙^) was evaluated. ABTS is a preformed stable organic radical with absorption maximum at 734 nm; nitric oxide (NO^∙^) is a pivotal proinflammatory mediator [31] and its contribution to oxidative damage is due to the reaction with superoxide to form the peroxynitrite anion, which is a potential strong oxidant that can decompose to produce ^∙^OH and NO_2_ [32]. In the present study, nitroprusside (SNP) was employed as a NO radical donor in the aim to evaluate the anti-inflammatory properties of native acid and acetylated hyaluronic acid. NO^∙^ released from SNP, indeed, has a strong NO^+^ character which can alter the structure and function of many cellular components. The scavenger ability of each sample (HA or HA-Acet) was evaluated in terms of radical reduction and data have been expressed as inhibition (%) and reported in Table 1. Both samples were found to have good and comparable scavenging properties towards the selected radicals confirming that the acetylation of native HA does not affect the biological activity of this polysaccharide.

### 3.2. Bioavailability Studies

Dialysis tubing procedure is a fast and low cost method to evaluate the bioavailability of different kinds of compounds and, in this study, it was used in the aim to evaluate the bioavailability of native HA and HA-Acet. Bioavailability was defined as the percentage of tested HA and HA-Acet recovered in the bioaccessible fraction, after* in vitro* digestion, in relation to the original nondigested samples. This value can be calculated by the following equation:

(2) $$\begin{array}{c} \left(\frac{\text{bioaccessible}\;\text{content}}{\text{total}\;\text{content}\;}\right)\times 100. \end{array}$$

In the present study, we supposed that the chemical modification of native HA by introducing acetyl groups can improve the bioavailability of this biopolymer. The obtained data (Table 1) confirmed our supposition showing that the acetylation of native polysaccharide increases its bioavailability of six times. This higher value could be ascribable to the presence of acetyl moieties which make the polymeric backbone more lipophilic.

### 3.3. *In Vitro* Cytotoxicity and Anti-Inflammatory Experiments

We next evaluated the* in vitro* cytotoxic activity of HA and HA-Acet on three different cell lines (J774.A1, WEHI-164, and HEK-293). Our results clearly showed a low cytotoxicity, compared to 6-MP, on all the three used cell lines and also at the concentration range (i.e., 12.5–100 *μ*mol/L) used for NO release determination in J774A.1 cell line; HA or HA-Acet treatments did not elicit antiproliferative effects, as evidenced by the IC_50_ results shown in Table 2.

**Figure 2:**
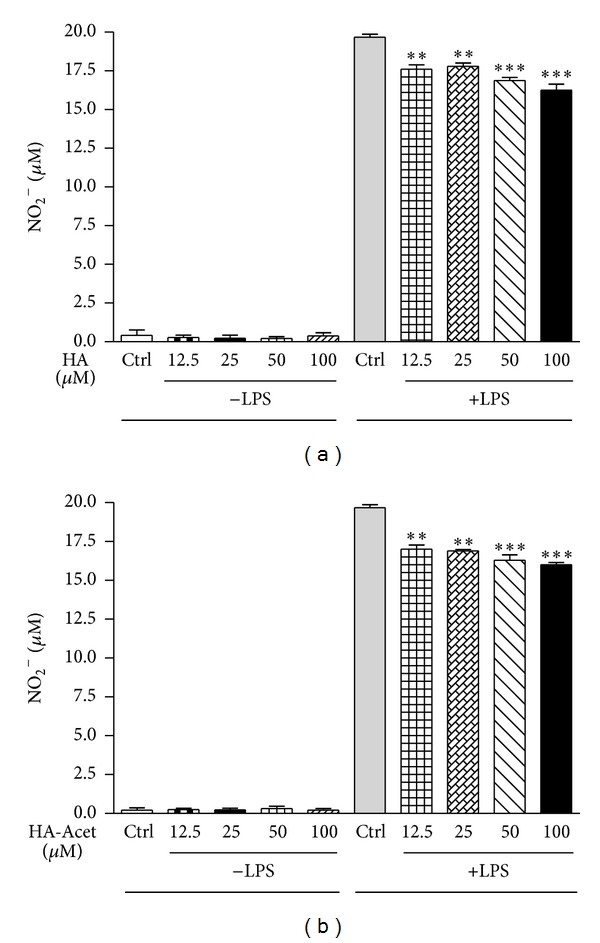
Effect of HA (Panel (a)) and HA-Acet (Panel (b)) on NO release from J774A.1 macrophages stimulated with LPS for 24 h. *** and ** denote *P* < 0.001 and *P* < 0.01, respectively, versus LPS alone.

The anti-inflammatory properties observed in previous experiments have been confirmed, as well, by testing HA and HA-Acet on J774A.1 murine macrophages. The latter were stimulated with LPS (1 *μ*g/mL), in the presence or absence of HA or HA-Acet (12.5–100 *μ*mol/L), to determine whether these compounds were able to modulate NO release. J774A.1 cells challenged with LPS exhibited a high increase of NO accumulation, evaluated as nitrite, as shown in Figure 2, panels (a)-(b), whereas HA or HA-Acet, per se, did not affect basal NO production at the tested concentrations (12.5–100 *μ*mol/L). Conversely, a significant reduction in NO release was detected in LPS-treated macrophages in presence of HA or HA-Acet at all tested concentrations, with a slight increase of HA-Acet ability in decreasing NO release from cells. We believe that this feature is most probably due to the better bioavailability and higher stability of HA-Acet with respect to the free HA form.

It is noteworthy that HA is usefully employed for the preparation of several derivatives which have been used as vector or delivery system for many molecules used in therapy mostly, but not only, for cancer treatment, due to the observation that HA-binding receptors such as cluster determinant 44 (CD44), receptor for hyaluronic acid-mediated motility (RAHMM), and lymphatic vessel endothelial receptor-1 (LYVE-1) are dramatically overexpressed in cancer cells [33–35]. Under this point of view, acetylation is a simple and suitable technique which allows increasing the hydrophobicity without impairing the ability of HA-receptors to interact with the acetylated-HA [36]. Moreover, it should be considered that generation of ROS (reactive oxygen species) plays a key role in human diseases and aging process and that HA is involved in the activation and modulation of the inflammatory response, including a scavenging activity towards ROS, such as hydroxyl radical (^∙^OH). On the other hand, inhibition of tumor cells and protection of tissue from free radical damage have also been attributed to a mixture of hyaluronic acid fragments and, in recent years, several reports described that HA exerts antiageing effect with potential antioxidant properties both* in vitro* and* in vivo *[37–39]. The efficacy ofthese considerable properties is related to many factors and, more strictly, to the catabolism of HA in the considered biologic environment. The major actors involved in its degradation are hyaluronidases, which would diminish its presence in the extracellular environment, so that the strategy to chemically modify HA (e.g., by acetylation), most importantly without altering the interaction with its receptors, has been pursued over time in order to increase its stability, bioavailability, and, lastly, its effects. Our results are promising for further studies addressed to a better understanding of the interactions of HA-Acet with biological molecules.

## 4. Conclusions

In this study we reported the synthesis of an acetylated HA derivative which exhibited a better bioavailability and stability with respect to the HA free form. These features have been confirmed, as well, by the evaluation of the NO release inhibition from murine monocyte/macrophage cell lines (J774.A1). HA-Acet showed a low cytotoxicity in all the three cell lines, at least at the drug doses used in the experiments and, moreover, a slight but significant increased anti-inflammatory activity, dose-dependent, has been evidenced. Our results bring a new contribution to the studies focused on the several biological properties and therapeutic uses of HA.

## Figures and Tables

**Table 1:** Antioxidant and anti-inflammatory activity and *in vitro *bioavailability of HA and HA-Acet. Statistical analysis was performed using Student's *t*-test.

Sample	Inhibition (%)		Bioavailability (%)
	ABTS	NO^•^	
HA	35 ± 0.7	77 ± 0.9	8 ± 0.7
HA-Acet	34 ± 1.0	75 ± 1.1	48 ± 0.3*

*indicates *P* < 0.001 of HA-Acet versus HA.

**Table 2:** The IC_50_, expressed as *μ*mol/L, value is the concentration of compound that affords a 50% reduction in cell growth (after a 24 h incubation). J774.A1 = murine monocyte/macrophage cell lines. HEK-293 = human epithelial kidney cell lines. WEHI-164 = murine fibrosarcoma cell lines. 6-MP = 6-mercaptopurine.

cmp	IC_50_ (*μ*M)		
	J774.A1	HEK-293	WEHI-164
HA-Acet	>100	>100	>100
HA	>100	>100	>100
6-MP	1	1.5	1.4
